# Introducing a model of cardiovascular prevention in Nairobi's slums by integrating a public health and private-sector approach: the SCALE-UP study

**DOI:** 10.3402/gha.v6i0.22510

**Published:** 2013-10-21

**Authors:** Steven van de Vijver, Samuel Oti, Thijs Cohen Tervaert, Catherine Hankins, Catherine Kyobutungi, Gabriela B. Gomez, Lizzy Brewster, Charles Agyemang, Joep Lange

**Affiliations:** 1 Health Challenges and Systems, African Population and Health Research Center, Nairobi, Kenya; 2 Department of Global Health, Amsterdam Institute for Global Health and Development, Academic Medical Centre, University of Amsterdam, Amsterdam, The Netherlands; 3 The Boston Consulting Group, Amsterdam, The Netherlands; 4 Department of Internal Medicine, Academic Medical Centre, University of Amsterdam, Amsterdam, The Netherlands; 5 Department of Vascular Medicine, Academic Medical Centre, University of Amsterdam, Amsterdam, The Netherlands; 6 Department of Public Health, Academic Medical Centre, University of Amsterdam, Amsterdam, The Netherlands

**Keywords:** hypertension, cardiovascular diseases, prevention, health system, slums, Africa

## Abstract

**Introduction:**

Cardiovascular disease (CVD) is a leading cause of death in sub-Saharan Africa (SSA), with annual deaths expected to increase to 2 million by 2030. Currently, most national health systems in SSA are not adequately prepared for this epidemic. This is especially so in slum settlements where access to formal healthcare and resources is limited.

**Objective:**

To develop and introduce a model of cardiovascular prevention in the slums of Nairobi by integrating public health and private sector approaches.

**Study design:**

Two non-profit organizations that conduct public health research, Amsterdam Institute for Global Health and Development (AIGHD) and African Population and Health Research Center (APHRC), collaborated with private-sector Boston Consulting Group (BCG) to develop a service delivery package for CVD prevention in slum settings. A theoretic model was designed based on the integration of public and private sector approaches with the focus on costs and feasibility.

**Results:**

The final model includes components that aim to improve community awareness, a home-based screening service, patient and provider incentives to seek and deliver treatment specifically for hypertension, and adherence support. The expected outcomes projected by this model could prove potentially cost effective and affordable (1 USD/person/year). The model is currently being implemented in a Nairobi slum and is closely followed by key stakeholders in Kenya including the Ministry of Health, the World Health Organization (WHO), and leading non-governmental organizations (NGOs).

**Conclusion:**

Through the collaboration of public health and private sectors, a theoretically cost-effective model was developed for the prevention of CVD and is currently being implemented in the slums of Nairobi. If results are in line with the theoretical projections and first impressions on the ground, scale-up of the service delivery package could be planned in other poor urban areas in Kenya by relevant policymakers and NGOs.

Cardiovascular disease (CVD) is the leading cause of mortality worldwide (1) with up to 80% of global CVD deaths occurring in low- and middle-income countries (LMICs) such as Kenya. By 2030, 2 million annual CVD deaths are expected in sub-Saharan Africa (SSA) (2).

The rise of CVDs in LMICs is driven mainly by globalization, industrialization, and urbanization (3), linked to an increased prevalence of CVD risk factors such as tobacco use, alcohol consumption, physical inactivity, and adoption of diets that are high in salt, sugar, and ‘unhealthy’ fat/oils.

The prevalence of behavioral and physiological risk factors for CVD is higher in urban than in rural areas (4). As the urban population in SSA is projected to increase from 395 million to 1.23 billion by 2050 (5), the burden of CVD in this region is bound to increase (6).

In Kenya, almost 70% of the urban population lives in slums or slum-like conditions where access to formal health services is limited (7). With existing healthcare services suffering from the ‘double burden of disease’ of endemic infectious diseases and emerging chronic diseases, CVDs are treated predominantly at late stages after complications have occurred (8). This makes care unnecessarily costly and less effective (8–10).

CVD is not only a public health problem, it is also an economic one (11). At the household and community levels, patients and their families often fall into poverty because of high healthcare-related expenditures (mostly out-of-pocket) for chronic conditions, as well as loss of income due to illness. At the macro level, losing young, productive people makes CVD a serious threat to the economies of SSA, an important issue considering that CVD deaths in SSA tend to occur 10 or more years earlier than in Europe and North America (12, 13).

Individual interventions for CVD prevention are both cost effective and scalable, even in resource-constrained settings (14–17). However, evidence is limited on cost-effective and sustainable community-based CVD prevention programs in LMICs in general (8, 18), and in severely resource-constrained settings such as slum settlements in particular.

The integration of public health and private-sector approaches to tackle CVD prevention could prove useful since, like CVD itself, prevention is closely linked to economic constraints. Such an approach could lead to the development of sustainable and scalable solutions that can be adapted locally to benefit public health in resource-poor settings in SSA.

The aim of this article is to describe a study design that integrates public health and private-sector approaches to lead to the development and introduction of a service delivery package for CVD prevention among urban poor in SSA.

## Study design

**Fig. 1:**
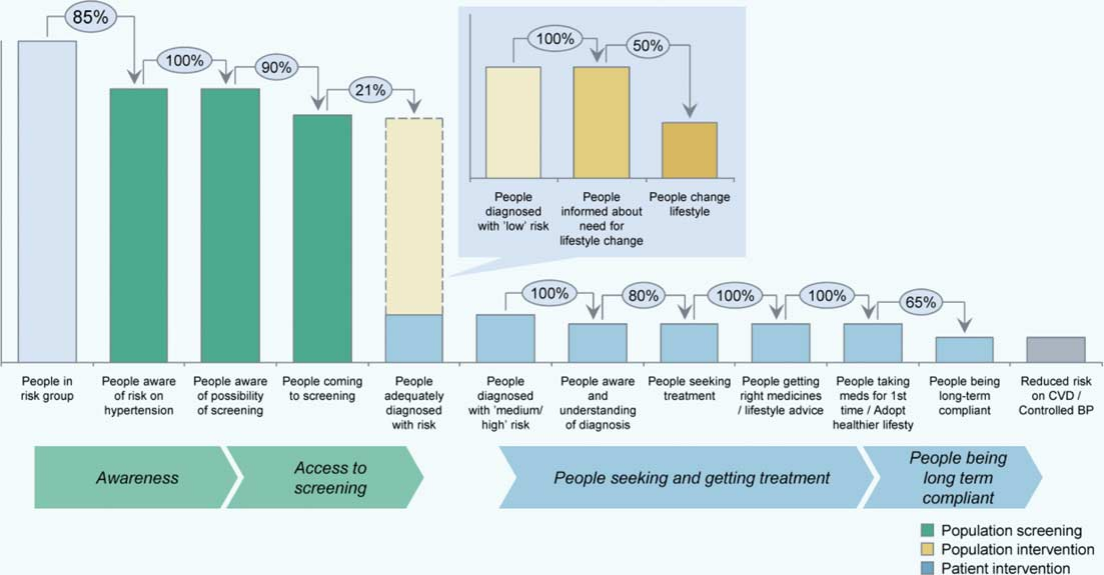
Cascade of care for hypertension and CVD risk reduction.

Two public health research organizations, the Amsterdam Institute for Global Health and Development (AIGHD) and the African Population and Health Research Center (APHRC), collaborated with a private-sector partner, Boston Consulting Group (BCG), to develop a service delivery package for primary prevention of CVD that is suitable for implementation in slum settings in Nairobi, Kenya. We were particular about developing a model that was at least in theory, cost effective. Previous studies in this setting (19) and results of an intervention project to improve patient access to treatment for hypertension and diabetes in primary care settings (APHRC, unpublished data), as well as a comprehensive literature review (20) informed the conceptual framework outlined in Fig. 1. This framework examines the flow of people from awareness of cardiovascular risk factors like hypertension and access to treatment, to adherence and successful blood pressure control. We show the main bottlenecks contributing to low service utilization and loss to follow-up, that is, becoming aware of CVD risk, accessing screening, seeking treatment, and complying with medication.

We constructed various alternatives of service delivery packages aimed at minimizing the bottlenecks identified in the theoretical framework (see Additional file 1). As evidence of community-based CVD prevention programs in LMICs is relatively limited, we borrowed important lessons from HIV prevention and control programs to address CVD (21). For instance, HIV control programs engage the community, involving fellow patients as peer educators and utilizing community health workers (CHWs) in home-based care, with demonstrated specific benefit to patient wellbeing and clinical outcomes (22). Overall, the cost and potential impact of the various alternative service delivery packages we considered were based on existing literature as well as our knowledge of the study area. The various alternative service delivery packages were then discussed with various CVD prevention stakeholders including policy makers, academic experts, program implementers, researchers, and field staff from previous projects, as well as local community representatives. Healthcare provision in Kenya is shared equally by the public and private sectors (23). Therefore, we aimed to include the feedback of representatives from both types of service providers. The objective was to ensure that each component of the service delivery package would address critical bottlenecks in the patient care continuum in a manner that is practical and acceptable within Nairobi slums.

Finally, the service delivery packages were ranked based on their theoretical cost effectiveness to determine the package most likely to succeed.

## Results

**Fig. 2:**
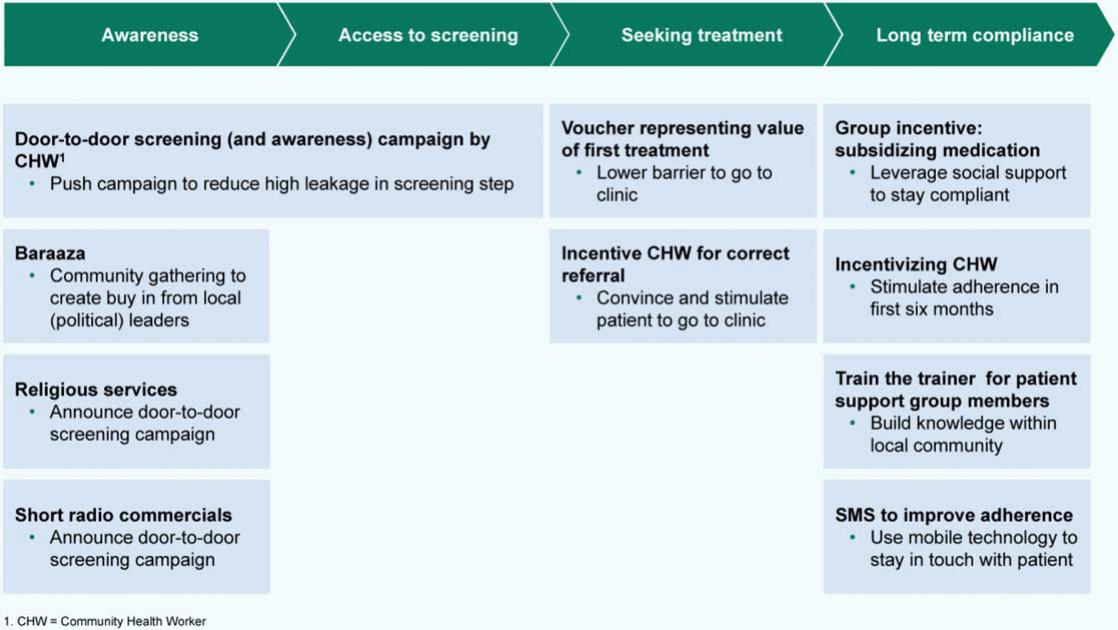
Components of the final service delivery package.

The outcome of the abovementioned process was the final selection of a service delivery package for primary prevention of CVD that comprised four elements: (i) increasing community awareness through announcements at community gatherings and religious services, and a local community radio jingle; (ii) improving access to screening for CVD risk factors such as hypertension through household visits; (iii) increasing treatment seeking through vouchers for free treatment and CHW incentives to follow up patients and persuade them to visit the clinic; and (iv) improving long-term compliance by setting up patient support groups, subsidizing medication through these groups, providing incentives for CHWs, and sending text messages (SMS) to remind patients of clinic appointments, medication use, and healthy lifestyles (Fig. 2). The final service delivery package for prevention of CVD has a specific focus on hypertension but it also includes other risk factors such as diabetes and obesity. This is because hypertension is the single most important risk factor for CVD (1).

For the package to remain potentially cost effective, a prioritization strategy is needed whereby only people aged 35 years and above would be screened. This population represents the group at highest risk: almost three-quarters of people with hypertension in the community are in this age category (APHRC, unpublished data).

**Fig. 3:**
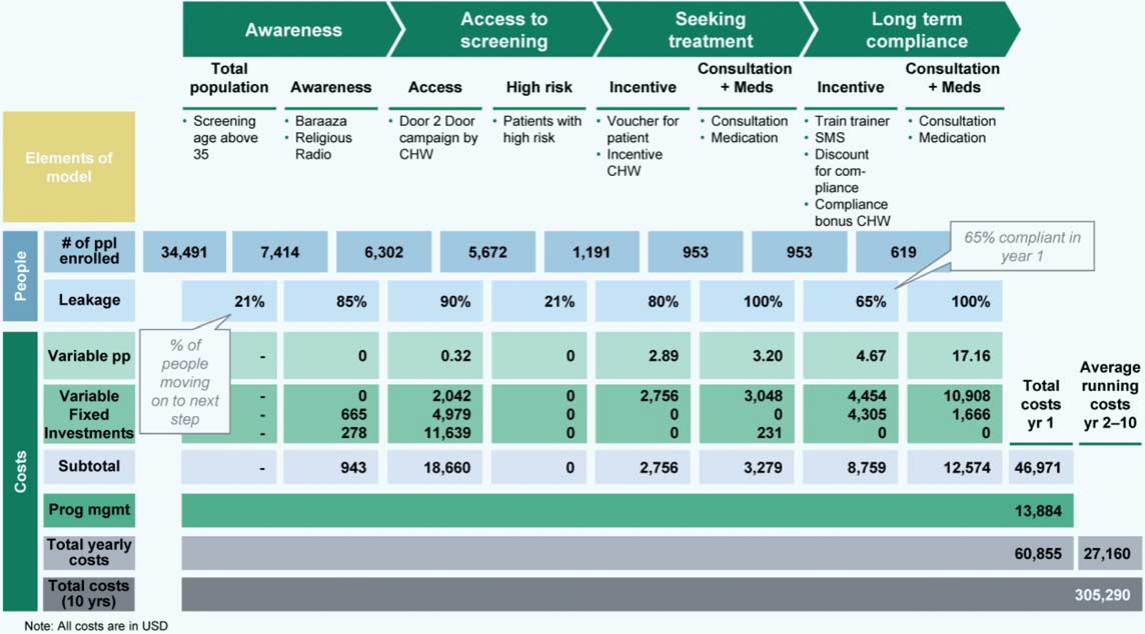
Potential cost calculations of the final service delivery package.

Overall, we estimated that the final selected service delivery package could avert 248–391 DALYs and cost less than one USD/person in the community (Fig. 3) resulting in a cost effectiveness of 760–1,200 USD/DALY averted. This makes the service delivery package, in theory, a highly cost-effective intervention for CVD risk prevention, with the potential to be sustainable in the resource-constrained settings.

The selected service delivery package began being implemented in August 2012 as the SCALE-UP Study (**S**ustainable model for **C**ardiovascular health by **A**djusting **L**ifestyle and treatment with **E**conomic perspective in settings of **U**rban **P**overty) in Korogocho, a Nairobi slum with a total population of 35,000. More than 100 CHWs and field interviewers have been trained during an intense 1-week training, after a pilot was completed. Staff have been recruited mainly from the local community with the assistance of the Ministry of Health who supplied us with a list of CHWs in the area. The training sessions were given by experienced APHRC staff and contained a wide range of topics such as clinical information on CVD and risk factors, techniques of screening and counselling, and ethical and practical aspects of the project. These led to an increase in knowledge among the participants from a pre-training score of 25/100 to a post-training score of 80/100). The CHWs are incentivized by receiving a fixed amount of money (approximately 3USD) for every person they screen and refer to the local clinic, and who demonstrates long-term compliance. The estimated total amount of payments and workload is in line with the guidelines for respectively compensation and duties of CHWs from the Ministry of Health. Therefore, introduction of the model in the existing public healthcare structure is feasible. Local clinic staffs were trained during a 1-day course on simple guidelines for treating people based on absolute cardiovascular risk, to narrow the treatment gap (24). These staff also showed a significant increase in knowledge with the pre-training score of 38/100 rising to 78/100 to post-training score. To ensure sustainability, these staffs are recruited mainly from existing healthcare services where they work as nurses and clinical officers. Because the clinics are open 2 days per week, they are able to adjust their existing work schedules for the rest of the week.

Enrolment of participants is ongoing, with close to 5,000 people 35 years and above already screened. This has led to approximately 900 referrals and 500 patients visiting the clinic. These numbers are close to the projected estimates. The population has reacted positively to the household screening with overall gratitude toward CHWs and low rates of refusals (3%). However, the fieldwork remains challenging due to the dynamic circumstances of the slum setting with high insecurity and mobility.

To measure the health impact of this initiative, two cross-sectional surveys (before and after, at least 1 year apart) will be conducted in Korogocho and the control site, Viwandani, a similar slum 10 km away. The usual standard of care for prevention and control of CVD in the control slum will be assessed. All resource utilization and related costs will be captured to assess the total cost of the intervention and estimate both affordability and feasibility. It is expected that the final evaluation of the project will reveal whether the service delivery package selected is cost-effective and acceptable.

From the study onset, we have maintained regular contact with key stakeholders, including the Ministry of Health, City Council of Nairobi, WHO, and leading non-governmental organizations (NGOs) such as Médecins sans Frontières. In order to facilitate potential scale-up to other settings, a manual is being developed to show how a similar package of interventions can be designed, implemented, and adapted to different contexts, should this intervention be proven cost effective.

## Conclusion

Through the collaboration of public health and private sector, a theoretically cost-effective model was developed for prevention of CVD, which is currently being implemented in a Nairobi slum. Collaboration between public health researchers and management consultants introduced innovative aspects to the design and selection of interventions. Based on early HIV screening approaches (25), public health researchers initially did not consider household screening as a realistic option. However, after discussions with the management consultants and a systematic comparison of different combinations of service delivery packages, door-to-door screening seemed likely to be more cost effective and sustainable within a comprehensive group of interventions than traditional stand-alone screening sites. The underpinning hypothesis is that active engagement of people is needed when products and projects, such as hypertension screening, are relatively unknown. Additionally, the household approach significantly reduces costs by combining awareness raising and screening, two activities that would otherwise be considered separately.

Performance-based payments and incentives as part of prevention and control strategies for CVD are relatively new in the public health sector in SSA. In low-resource settings, they may play an important role in making effective use of limited resources. The downside is that rigorous follow-up is required to prevent beneficiaries and program staff from manipulating the incentive system, especially in settings of extreme poverty such as slums. Furthermore, we experienced some resistance from key stakeholders such as CHWs to the idea of an incentive-based payment, preferring the old system of fixed remuneration.

If results are in line with the theoretical projections and first impressions on the ground, scale-up of the service delivery package could be extended to other poor urban areas in Kenya by relevant policymakers and NGOs. In time, this approach may also prove to be sustainable and scalable elsewhere in Africa.
